# Spatial distribution and factors associated with coexisting undernutrition among under-five children in Ethiopia: Evidence from the 2019 Mini-Demographic and Health Survey

**DOI:** 10.1371/journal.pone.0329750

**Published:** 2025-08-08

**Authors:** Meklit Melaku Bezie, Getayeneh Antehunegn Tesema

**Affiliations:** 1 Department of Public Health, Institute of Public Health, College of Medicine and Health Sciences, University of Gondar, Gondar, Ethiopia; 2 Department of Epidemiology and Biostatistics, Institute of Public Health, College of Medicine and Health Sciences, and Comprehensive Specialized Hospital, University of Gondar, Gondar, Ethiopia; Wachemo University, ETHIOPIA

## Abstract

**Background:**

Undernutrition remains a critical public health issue in Ethiopia, driving high under-five morbidity and mortality. Coexisting forms; stunting, wasting, and underweight magnify these risks but their spatial patterns and determinants remain poorly understood. This study investigates the geographic distribution and key factors of coexisting undernutrition among Ethiopian children under five to inform targeted, geographic-specific interventions.

**Methods:**

We conducted a secondary data analysis of the 2019 Ethiopia Mini Demographic and Health Survey (EMDHS), including a weighted sample of 4,952 children under five years of age. Spatial analysis was employed to explore the geographic distribution of coexisting forms of undernutrition, and significant spatial clusters were identified using SaTScan version 10. To examine associated factors, a multilevel binary logistic regression model was fitted. Variables with a p-value < 0.2 in the bivariable analysis were included in the multivariable model. Effect measures were reported using Adjusted Odds Ratios (AORs) with 95% Confidence Intervals (CIs).

**Results:**

The national prevalence of coexisting forms of undernutrition among children under five in Ethiopia was 19.6% (95% CI: 18.5,20.7), with marked regional disparities ranging from 5.2% in Addis Ababa to 30.7% in the Afar region. Spatial analysis identified a significant high-risk cluster spanning Afar, Amhara, Tigray, Benishangul-Gumuz, and northern Somali regions (Log-Likelihood Ratio [LLR] = 38.83, p < 0.001), indicating pronounced geographic heterogeneity. Maternal education at primary (AOR = 0.81, 95% CI: 0.68, 0.98), secondary (AOR = 0.53, 95% CI: 0.37, 0.77), and higher levels (AOR = 0.29, 95% CI: 0.17, 0.51), higher household wealth (richer: AOR = 0.68, 95% CI: 0.50, 0.92; richest: AOR = 0.53, 95% CI: 0.35, 0.80), and female sex (AOR = 0.80, 95% CI: 0.69, 0.92). Conversely, multiple births (AOR = 2.06, 95% CI: 1.33, 3.18) and residing in communities with high poverty levels (AOR = 1.44, 95% CI: 1.11, 1.87) significantly increased the risk of coexisting undernutrition.

**Conclusion:**

Significant geographic disparities in coexisting undernutrition among Ethiopian children under five highlight urgent hotspots in Afar, Amhara, Tigray, Benishangul, and northern Somali regions. Protective factors such as maternal education, household wealth, and female sex, while multiple births and poverty were risk factors. These findings highlight the need for geographically targeted interventions focused on hotspot areas, with an emphasis on improving maternal education and alleviating poverty to reduce coexisting forms of undernutrition and enhance child survival.

## Background

Child undernutrition remains a major public health concern in many Low- and Middle-Income Countries (LMICs) [1]. Each year, it tragically contributes to the deaths of approximately 5.2 million children in developing nations [2]. Undernutrition is primarily defined in three forms: wasting, stunting, and underweight [3]. Wasting is characterized by a low weight-for-height ratio, while stunting is identified through a low height-for-age measurement [4]. Underweight refers to a low weight-for-age ratio [5].

According to the World Health Organization (WHO), the global statistics on child malnutrition are alarming. In 2022, 149 million children were stunted, 45 million experienced wasting, and 37 million were underweight [6]. Additionally, UNICEF reports that nearly 40% of stunted children and over 25% of wasted children reside in Africa, including Ethiopia, which bears more than half of the global burden of undernutrition [7]. The 2019 Ethiopian Mini Demographic and Health Survey (EMDHS) further underscores the severity of the issue, revealing that over one-third of children under five are stunted and underweight, and 15% suffer from wasting [8].

In developing countries such as Ethiopia, children often experience multiple forms of undernutrition simultaneously due to limited access to food, healthcare, and sanitation [9]. In Ethiopia alone, 5.8% of children under the age of five suffer from these overlapping forms of undernutrition [10]. The risk of mortality among children with multiple coexisting types of undernutrition is alarmingly high, with a likelihood 12.75 times greater than those without [11]. Research suggests that nearly half of child mortality cases are associated with coexisting forms of undernutrition. Undernutrition can have severe and long-lasting effects, particularly in young children [12,13]. This issue is critical, as it contributes to 44.8% of acute respiratory tract infections and 27.96% of diarrheal cases among children under five [14]. Early childhood is a crucial period for brain development, and inadequate nutrition significantly impacts cognitive growth, overall development, academic performance, and social interactions [15].

Research suggests that the coexistence of various forms of undernutrition arises from a complex interplay of factors [16,17]. The primary underlying causes include infectious diseases, environmental conditions, inadequate nutrition, and suboptimal infant feeding and caregiving practices [18]. Several factors influence these practices, including maternal education [19,20], the child’s age [21,22], maternal age at childbirth [23], place of residence [24], parental employment status [25,26], birth order [27], Antenatal Care (ANC) [28], maternal Body Mass Index (BMI) [29,30] and household wealth status [31]. Addressing these determinants through targeted interventions and policies is essential to effectively reduce undernutrition and improve the overall health and well-being of children and families.

In Ethiopia, increasing emphasis has been placed on nutrition-sensitive interventions to prevent undernutrition and achieve the Sustainable Development Goals (SDGs) [32]. This approach aligns with global nutrition targets aimed at improving health outcomes [33]. Specifically, SDG 2 and SDG 3 aim to end all forms of malnutrition by 2030, with a particular focus on reducing stunting and wasting among children under five. These global goals are intended to guide and influence national policies and strategies, promoting integrated efforts to improve the overall health and well-being of children and families. However, a major challenge in the country is the coexistence of multiple forms of undernutrition within the same child [34]. Additionally, significant regional disparities exist in the prevalence of undernutrition, with considerable geographic variation in both its forms and severity across different regions [35].

Despite the public health importance of this, limited research has explored the spatial distribution of coexisting forms of undernutrition and the factors contributing to these geographic disparities. This study seeks to fill that gap by analyzing the spatial patterns of coexisting undernutrition and identifying key determinants influencing its distribution among children under five. Using data from the 2019 Ethiopian Mini-DHS, this study aims to highlight regional disparities and underscore the value of spatial analysis in guiding targeted, evidence-based interventions in the most affected areas.

## Methods and materials

### Study setting, design, and period

A community-based cross-sectional study was conducted based on data from the 2019 Ethiopian Mini Demographic and Health Survey (EMDHS), conducted between March and June 2019. Ethiopia is an East African country located in the Horn of Africa. It comprises nine regions (Afar, Amhara, Benishangul-Gumuz, Gambella, Harari, Oromia, Somali, Southern Nations, Nationalities, and People’s Region (SNNPR), and Tigray) and two administrative cities (Addis Ababa and Dire Dawa). More than 80% of the country’s total population resides in the regional states of Amhara, Oromia, and SNNPR [36].

The EMDHS employed a stratified two-stage cluster sampling technique, with Enumeration Areas (EAs) and households serving as the primary and secondary sampling units, respectively. A detailed description of the sampling procedure is available in the full EMDHS 2019 report [37]. In the first stage, a total of 305 EAs (93 in urban areas and 212 in rural areas) were selected using probability proportional to the EA size. In the second stage, an average of 30 households per cluster were selected. For this study, we used the Kids Record (KR) dataset, analyzing a weighted sample of 4,952 children under the age of five.

### Study variables

The dependent variable in this study was the coexisting forms of undernutrition among children under five. It was defined based on children’s height-for-age (HAZ), weight-for-height (WHZ), and weight-for-age (WAZ). Stunting was classified as HAZ < −2 standard deviations, wasting as WHZ < −2 standard deviations, and underweight as WAZ < −2 standard deviations. A coexisting form of undernutrition was defined as the presence of more than one form of undernutrition (i.e., stunting, wasting, or underweight) in a child [38].

The independent variables included the child’s sex (male or female), maternal age (15–24, 25–34, and ≥35 years), maternal educational status (no formal education, primary, secondary, or higher), and household wealth status (poorest, poorer, middle, richer, or richest). Other variables included the sex of the household head (male or female), marital status (married, never in union, divorced, widowed, living with a partner, or separated/no longer living with a partner), and child age in months (<12, 12–23, 24–35, and 36–59). Additionally, household environmental and maternal health factors were considered, including water source (improved or unimproved), toilet facility (improved or unimproved), place of delivery (health facility or home), and mode of delivery (cesarean or vaginal). Reproductive factors included preceding birth interval (primiparous, < 2 years, or ≥2 years), parity (primiparous, multiparous, or grand multiparous), and the number of gestations (singleton or multiple).

In MEDHS data no variable describes the cluster except region and place of residence. Therefore, individual-level variables were aggregated at the cluster level to generate community-level variables to see whether cluster-level variables had an effect on coexisting forms of undernutrition and which were categorized by their proportion as higher or lower based on national median value since it was not normally distributed (community women education and community poverty). Community-level variables used in the analysis were from two sources; direct community-level variables that were used without any manipulation and aggregated community-level variables that were generated by aggregating individual-level variables at the cluster level.

### Statistical analysis

#### Spatial analysis.

Arc GIS version 10.7 and SaTScan software version 10 were used for visualization, hotspot detection, spatial autocorrelation, interpolation and to detect the high likelihood of coexisting forms of undernutrition.

#### Spatial autocorrelation analysis.

Spatial autocorrelation is a spatial analysis that quantifies the degree to which a variable correlates with itself across different geographic. This concept underpins the idea that states that close entities tend to share similar characteristics and values than far apart. Moran’s I is a special statistic used to measure the spatial relationship of variables across areas with a single value. The range of Moran’s I is from −1–1, where a value close to −1 indicates that coexisting form of undernutrition is dispersed. Conversely, a value close to +1 tells us that the coexisting form of undernutrition is clustered and randomly distributed if the value is zero. To assess the statistical significance of the observed spatial pattern p value was used. A low p-value (less than 0.05) suggests that the observed pattern is unlikely to be due to random variation.

#### Hot spot analysis (Getis-OrdGi* statistic).

The Getis-Ord Gi* statistic was computed to assess spatial autocorrelation variations across the study area by calculating the Gi* value for each region. The Z-score was used to determine the statistical significance of clustering, while the p-value indicated the level of significance. A high Gi* value signifies a “hotspot,” whereas a low Gi* value indicates a “cold spot.” [39].

#### Spatial interpolation.

A statistical technique used to estimate the unsampled location of Ethiopia of the coexisting form of undernutrition among under-five based sampled data, the spatial interpolation technique was used. We used the Bayesian kriging method which accounts for geographical proximity and similarity of data points and also incorporates the inherent uncertainty associated with the estimate. Furthermore, the Bayesian takes account weights into the spatial arrangement of all observations.

#### Spatial scan statistical analysis.

Spatial scan statistical analysis was conducted using the Bernoulli model to identify the most probable clusters of coexisting forms of undernutrition among under-five children. This analytical approach involved the application of a scanning window that traversed various spatial dimensions, allowing for the examination of geographic patterns in the prevalence of undernutrition. A p-value was assigned to each identified cluster to assess its statistical significance. Kuldorff’s SaT Scan version 10 software was used for the analysis. Under-five children with coexisting forms of undernutrition were taken as cases and those who did not experience coexisting forms of undernutrition as controls to fit the model. The default maximum spatial cluster size of < 50% of the population was used as an upper limit since it allowed both small and large clusters to be detected and ignored clusters that contained more than the maximum limit. Choosing a cluster size of 50% of the total population is the default option for the maximum size of the scan window. It is often used to search for clusters that are most likely to have a higher likelihood value.

Likelihood and p-value were used to determine if the number of coexisting undernutrition cases within potential clusters was significantly higher than expected for each potential cluster. The scanning window that demonstrated the highest likelihood emerged as the most probable performing cluster within the analysis. To assess the statistical significance of each identified cluster, a p-value was assigned through a rigorous process known as Monte Carlo hypothesis testing. This method involved a systematic comparison of the rank of the maximum likelihood estimate derived from the actual dataset against the ranks obtained from a series of randomly generated datasets.

#### Multilevel binary regression analysis.

Given the complex survey design of the EMDHS data, under-five children were nested within clusters (EAs). Consequently, children within the same cluster may share similar characteristics, potentially violating the fundamental assumptions of standard regression models, such as the assumption of equal variance and independence. Therefore, a multilevel binary logistic regression model was employed, incorporating both fixed effects and random effects. The presence of clustering was assessed using the Intra-class Correlation Coefficient (ICC), the Likelihood Ratio (LR) test, and the Median Odds Ratio (MOR). Model comparison and fitness were conducted using log-likelihood and deviance. Four models were constructed. The first model (null model) was a random-intercept model without any predictors, used to estimate the baseline variation in coexisting undernutrition across clusters. The second model included only individual-level variables. The third model adjusted for community-level variables, while the fourth model was fitted with both individual-level and community-level variables simultaneously. The fourth model having the lowest deviance (−Log-Likelihood Ratio (−2LLR)) was selected as the best-fitting model.

Variables with a p-value <0.2 in the bivariable analysis were included in the multivariable mixed-effects binary logistic regression model. In the final model, an Adjusted Odds Ratio (AOR) with a 95% Confidence Interval (CI) and a p-value <0.05 was used to identify statistically significant associations with coexisting forms of undernutrition.

### Ethics

The EDHS survey adhered to all required ethical clearance protocols. Specifically, the 2019 EMDHS received ethical approval from the Ethiopian Health and Nutrition Research Institute Review Board, the Ministry of Science and Technology, the Centers for Disease Control and Prevention (CDC), and ICF International’s Institutional Review Board. Written informed consent or assent for minors—was obtained from all participants or their caregivers prior to data collection. The study strictly followed the ethical standards outlined in the 1964 Declaration of Helsinki.

## Results

### Descriptive results

A total of 4952 children under five were included. The majority of the children were from the Oromia region (39.90%) followed by the Amhara region (19.22%). About 3712 (74.96%) of the children belonged to rural settings. About 1753 (35.39%) and 365 (7.36%) of the children were born to mothers who attained primary and secondary education, respectively. Of the total 4952, 2432 (49.11%) of the children were born at health facilities with 5.17% of them being through caesarean delivery. About 1983 (40.05%) of children aged 36 months and above, and more than half (54.00%) were born to mothers aged 25–34 years (Table 1).

**Table 1:** The socio-demographic and background characteristics of the study participants in Ethiopia based on the 2019 mini-DHS.

Variable	Weighted frequency	Percentage (%)
**Region**		
Tigray	350	7.08
Afar	74	1.49
Amhara	952	19.22
Oromia	1976	39.90
Somalia	339	6.85
Benishangul-gumuz	58	1.18
SNNPR	1003	20.26
Gambella	21	0.43
Harari	14	0.29
Dire-Dawa	138	2.79
Addis Ababa	26	0.52
**Residence**		
Urban	1240	25.04
Rural	3712	74.96
**Maternal education**		
No education	2654	53.59
Primary	1753	35.39
Secondary	365	7.36
Higher	181	3.66
**Household wealth status**		
Poorest	1137	22.96
Poorer	1093	22.07
Middle	930	18.79
Richer	874	17.64
Richest	918	18.53
**Child’s age (in months)**		
< 12	994	20.08
12–23	996	20.10
24–35	979	19.77
≥ 36	1983	40.05
**Maternal age (in years)**		
15–24	1134	22.90
25–34	2674	54.00
≥ 35	1144	23.10
**Sex of household head**		
Male	4,312	87.07
Female	640	12.93
**Sex of child**		
Male	2521	50.90
Female	2431	49.10
**Number of gestations**		
Singleton	4844	97.82
Multiple	108	2.18
**Parity**		
Primiparous	742	14.98
Multiparous	2840	57.35
Grand multiparous	1370	27.67
**Water source**		
Unimproved	1709	34.52
Improved	3243	65.48
**Toilet facility**		
Unimproved	4111	83.01
Improved	841	16.99
**Place of delivery**		
Home	2520	50.89
Health facility	2432	49.11
**ANC**		
No	884	17.86
Yes	2707	54.67
Missing	1361	27.48
**Mode of delivery**		
Vaginal	4696	94.83
Caesarean delivery	256	5.17
**Preceding birth interval**		
< 2 years	810	16.36
> = 2 years	3071	62.02
Primiparous	1071	21.62
**Marital status**		
Never in union	18	0.36
Married	4671	94.32
Living with partner	34	0.68
Widowed	58	1.17
Divorced	122	2.45
No longer living together/separated	50	1.01
**Stunted**		
No	3105	62.70
Yes	1847	37.30
**Wasted**		
No	4597	92.83
Yes	355	7.17
**Underweight**		
No	3903	78.82
Yes	1049	21.18
**Coexisting form of undernutrition**		
No	3981	80.39
Yes	971	19.61

### Prevalence of coexisting forms of undernutrition

**Fig 1:**
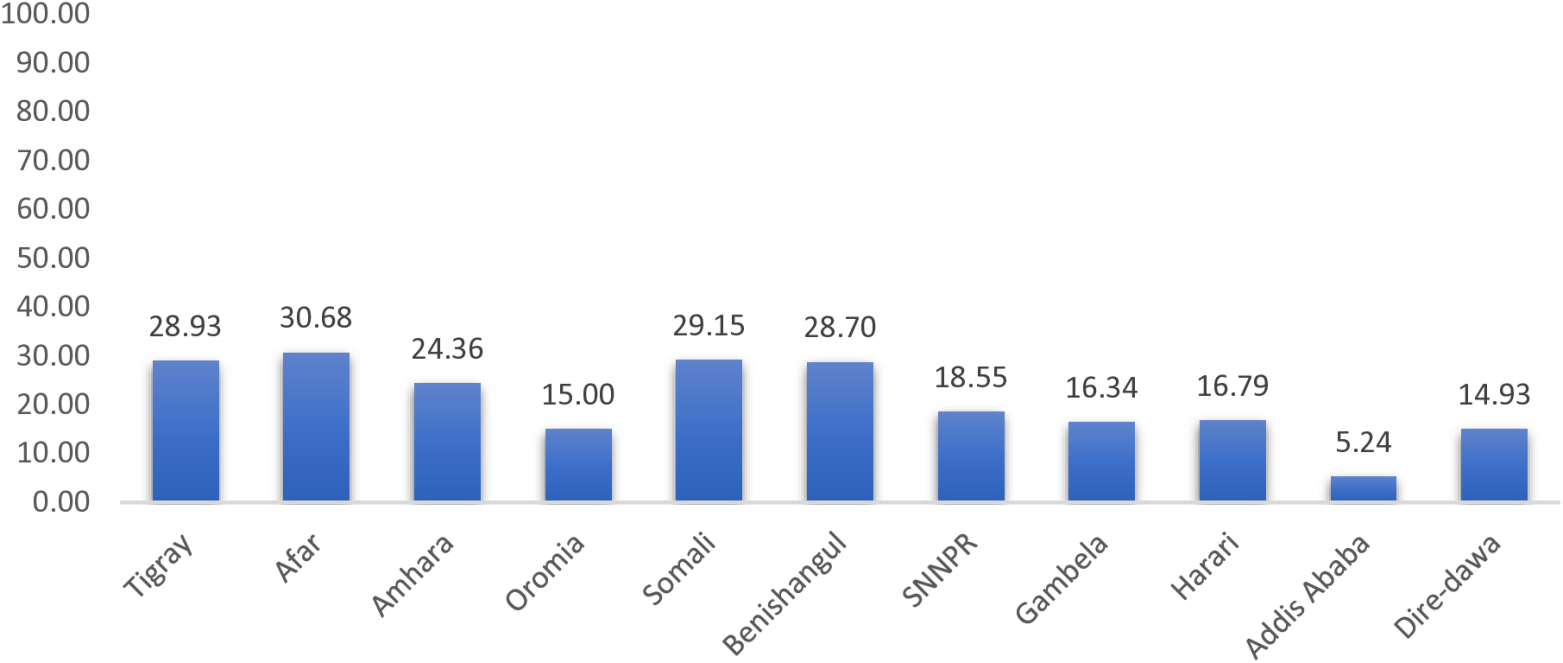
The prevalence of coexisting form of undernutrition among children under-five in Ethiopia.

The prevalence of a coexisting form of undernutrition among under-five children in Ethiopia was 19.61% (95% CI: 18.52, 20.74). The prevalence varied across regions ranging from 5.24% in Addis Ababa to 30.68% in the Afar region (Fig 1).

### Spatial analysis results

#### Spatial distribution of coexisting forms of undernutrition.

**Fig 2:**
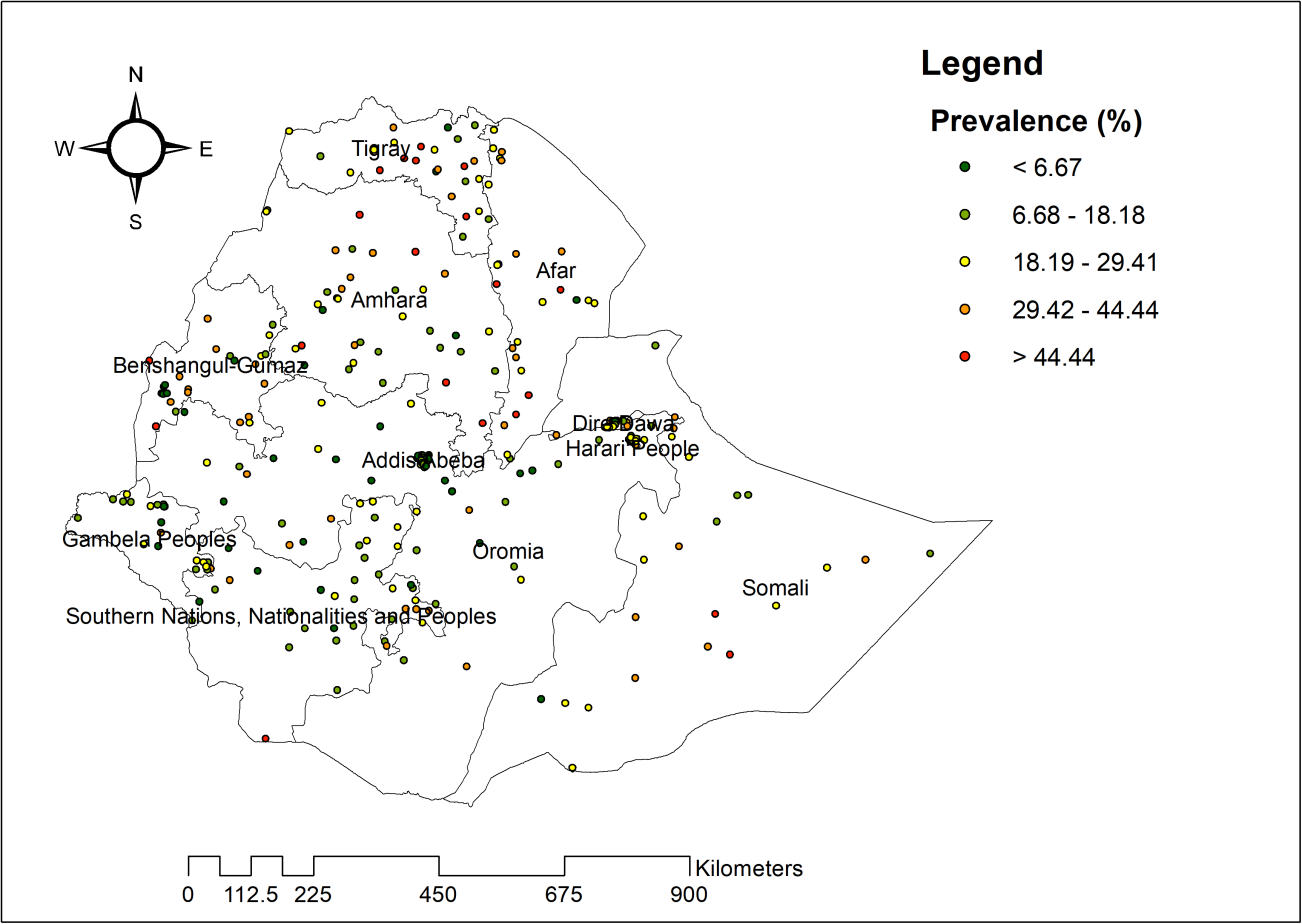
The spatial distribution of coexisting form of undernutrition among under-five children in Ethiopia, 2019.

In our analysis, we examined 305 clusters to assess the spatial distribution of coexisting forms of undernutrition across Ethiopia. The map displays EAs within these clusters, with each point representing the prevalence of coexisting forms of undernutrition. Areas marked in red indicate high prevalence, while green areas reflect lower prevalence rates. Regions with notably higher rates of coexisting undernutrition include central and southeastern Tigray, southwestern Benishangul-Gumuz, eastern Gambella, central and southwestern Amhara, southwestern Afar, and both southwestern and northeastern parts of the Somali region. In contrast, lower proportions of coexisting undernutrition were observed mainly in Gambella, SNNPR, western Amhara, and northeastern Tigray (Fig 2).

**Fig 3:**
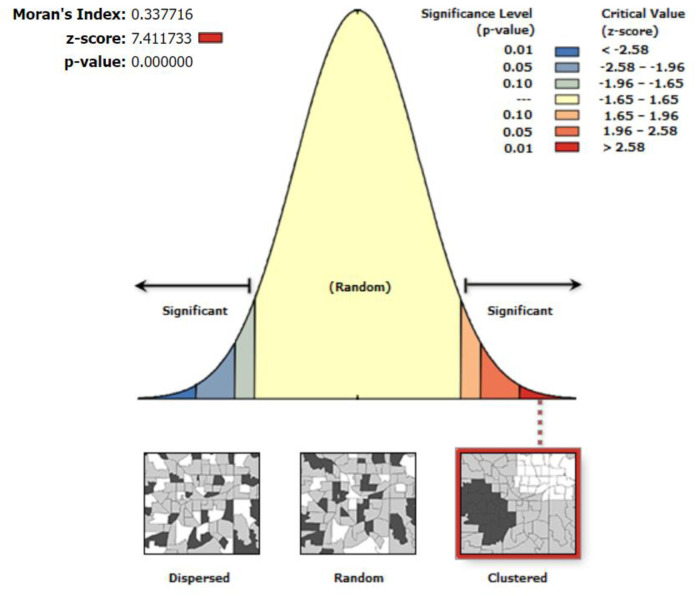
Global Moran’s index statistical analysis of spatial autocorrelation of coexisting form of undernutrition among under-five children in Ethiopia.

Our findings revealed that the spatial distribution of coexisting forms of undernutrition in Ethiopia is not random. This is supported by a Global Moran’s I value of 0.337 (p < 0.001), indicating a statistically significant spatial autocorrelation (Fig 3). The cluster analysis (right panel) demonstrates that regions with high rates of coexisting undernutrition are spatially concentrated across the study area. A z-score of 7.4 suggests a strong clustering pattern, indicating that areas with similar levels of undernutrition are more geographically concentrated than would be expected by chance. The bright red and blue areas at the ends of the spectrum denote statistically significant clusters (Fig 3).

**Fig 4:**
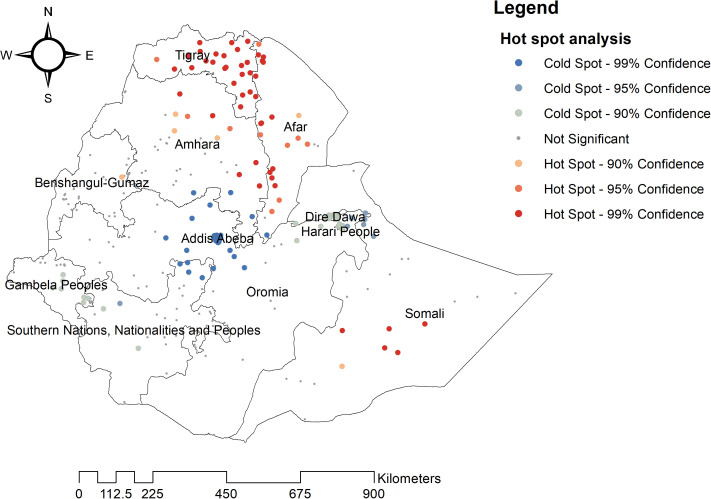
Hotspot analysis of coexisting form of undernutrition among under-five children in Ethiopia.

Hotspots of coexisting undernutrition marked in red were predominantly found in central, eastern, and southern Tigray; western and southern Afar; central, northern, and southeastern Amhara; and the central Somali region. Conversely, areas marked in blue and light green represent significant non-risk zones, or cold spots, located in Addis Ababa, central Oromia, Dire Dawa, Harari, and eastern SNNPR. Notably, as the level of confidence increases, the statistical significance of these spatial patterns also strengthens (Fig 4).

### Spatial interpolation of coexisting form of undernutrition

**Fig 5:**
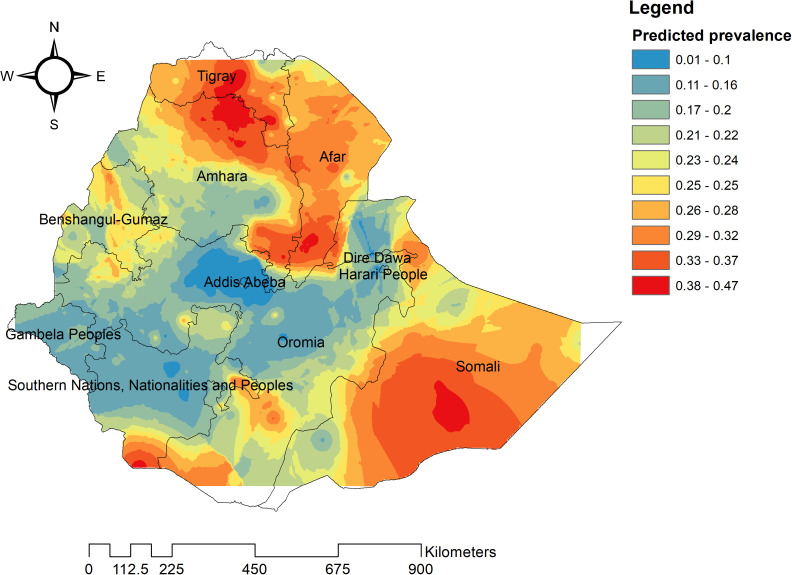
Spatial interpolation of coexisting form of undernutrition among under-five children in Ethiopia.

Our analyses have identified central and southern Tigray, southwest Afar, southwest Oromia, northern and eastern Amhara, and eastern Benishangul, along with Somali and southern SNNPR, as regions particularly susceptible to coexisting forms of undernutrition. In contrast, areas predicted to have lower risk levels include Oromia, Addis Ababa, and Gambella. By employing the Empirical Bayesian Kriging interpolation method, continuous imagery was generated to illustrate the distribution of coexisting forms of undernutrition and associated factors among under-five children (Fig 5).

### Spatial scan statistical analysis

**Fig 6:**
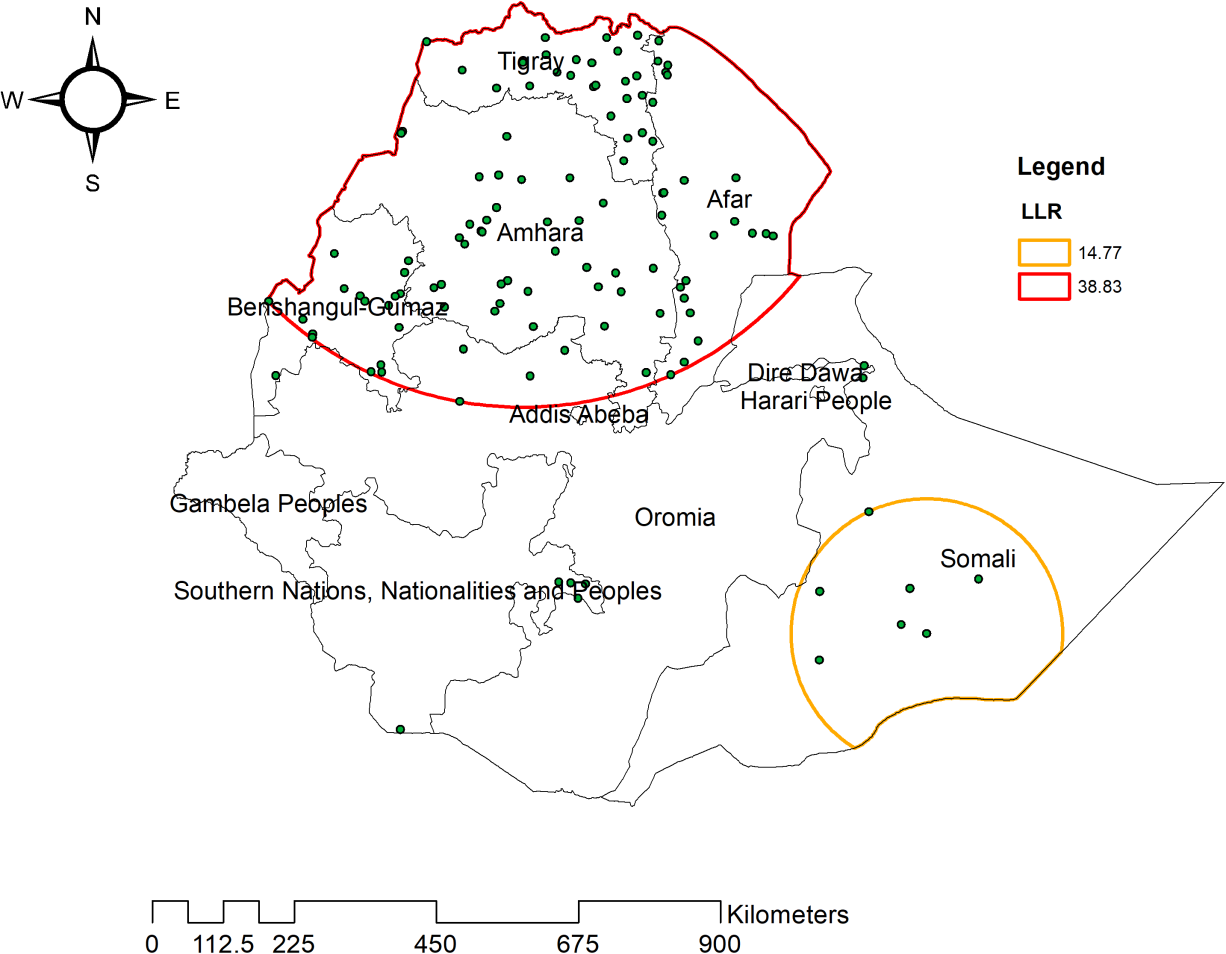
SaTScan analysis of hotspot areas of undernutrition among under-five children in Ethiopia, 2019.

The spatial scan statistical analysis identified a total of six clusters. Among these, the primary cluster was located in the Tigray, Amhara, Benishangul-Gumuz, Afar regions, and the northern border of Addis Ababa, with its center at coordinates (13.987653 N, 37.973902 E) and a radius of 545.08 km. This cluster had a Relative Risk (RR) of 1.6 and a Log-likelihood Ratio (LLR) of 38.8, with a p-value less than 0.001. This indicates that under-five children within this spatial window were 1.6 times more likely to experience coexisting forms of malnutrition compared to those outside the identified window. A secondary cluster was located in the Somali region, centered at (5.856584 N, 43.726016 E) with a radius of 213.70 km. The bright red circular window in the analysis represents statistically significant spatial windows with a high likelihood of coexisting forms of undernutrition (Table 2 & Fig 6).

**Table 2:** Sat Scan analysis of coexisting forms of undernutrition among under-five children in Ethiopia.

Cluster	Enumeration area identified	Coordinated/radius	Population	Case	RR	LLR	p
1	8,1,9,6,7,22,13,12,21,11,2,14,56,10,16,4,23,5,55,17,85,18,27,37,29,53,60,45,44,72,66,52,33,77,49,161,68,160,168,40,150,169,3,15,82,83,25,84,78,20,35,36,39,24,38,57,19,62,58,59,61,74,54,81,75,46,65,70,76,167,71,63,79,51,34,162,80,64,163,30,73,148,159,47,48,67,166,31,158,100,164,119,26,50,32,99,43,69,167,149,156,42,98,146	(13.987653 N, 37.973902 E)/545.08 km	1820	528	1.60	38.83	<0.01
2	137,138,123,135,142,136,145	(5.856584 N, 43.726016 E)/213.7 km	152	63	1.93	14.77	<0.01

### Individual and community-level determinants of coexisting forms of undernutrition

#### The random effect analysis result.

Model comparisons using deviance (−2LL) and the Likelihood Ratio (LR) test confirmed that the mixed-effects binary logistic regression model provided a significantly better fit than the standard model (LR test: chi²(01) = 129.6, p < 0.01). Among the four fitted models, the final model (including both individual- and community-level variables) had the lowest deviance.

The ICC decreased from 12.96% in the null model to 5% in the final model, indicating reduced between-cluster variability after adjustment. The Median Odds Ratio (MOR) also declined from 1.70 to 1.51, suggesting persistent but reduced cluster-level heterogeneity. The Proportional Change in Variance (PCV) reached 61.7%, showing that the included covariates explained about 62% of the cluster-level variation in coexisting undernutrition.

#### The fixed effect analysis result.

Variables such as residence, maternal education, household wealth status, number of gestations, sex of the child, parity, toilet facility, water source, place of delivery, marital status, community maternal education, community poverty, and region had a p-value < 0.2 in the bivariable multilevel binary logistic regression analysis and were therefore included in the multivariable multilevel binary logistic regression model. In the multivariable analysis, maternal education, number of gestations, sex of the child, household wealth status, region, and community wealth status were significantly associated with coexisting forms of undernutrition among children under five.

The odds of coexisting forms of undernutrition were reduced by 19% (AOR = 0.81, 95% CI: 0.68, 0.98), 47% (AOR = 0.53, 95% CI: 0.37, 0.77), and 71% (AOR = 0.29, 95% CI: 0.17, 0.52) among children whose mothers attained primary, secondary, and higher education, respectively, compared to children of mothers with no formal education. Children from richer and richest households had 32% (AOR = 0.68, 95% CI: 0.50, 0.92) and 47% (AOR = 0.53; 95% CI: 0.35, 0.80) lower odds of coexisting undernutrition, respectively, compared to those from the poorest households. Children from multiple births had more than twice the odds of experiencing coexisting undernutrition (AOR = 2.06, 95% CI: 1.33, 3.18) compared to singletons. Female children had 20% lower odds of coexisting forms of undernutrition (AOR = 0.80, 95% CI: 0.69, 0.92) compared to males.

Regarding regional variation, children residing in Oromia (AOR = 0.35, 95% CI: 0.23, 0.51), Somali (AOR = 0.55, 95% CI: 0.36, 0.84), Benishangul-Gumuz (AOR = 0.64, 95% CI: 0.43, 0.97), SNNPR (AOR = 0.48, 95% CI: 0.33, 0.71), Gambella (AOR = 0.37, 95% CI: 0.24, 0.59), Harari (AOR = 0.52, 95% CI: 0.33, 0.81), Addis Ababa (AOR = 0.21, 95% CI: 0.11, 0.42), and Dire-Dawa (AOR = 0.45, 95% CI: 0.28, 0.72) had significantly lower odds of coexisting undernutrition compared to children in Tigray. Moreover, children from communities with high poverty levels had increased odds of coexisting forms of undernutrition (AOR = 1.44; 95% CI: 1.11, 1.87) (Table 3).

**Table 3:** Multilevel binary logistic regression analysis of factors associated with coexisting form of undernutrition among under-five children in Ethiopia.

Variable	Null model AOR (95% CI)	Model 1 AOR (95% CI)	Model 2 AOR (95% CI)	Model 3 AOR (95% CI)
**Individual level factors**				
**Maternal educational status**				
No education		1		1
Primary		0.78 (0.65,0.94)		0.81 (0.68, 0.98)^**^
Secondary		0.52 (0.36, 0.74)		0.53 (0.37, 0.77)^**^
Higher		0.29 (0.17, 0.51)		0.29 (0.17, 0.52)^**^
**Household wealth status**				
Poorest		1		1
Poorer		0.84 (0.68, 1.04)		0.94 (0.75, 1.17)
Middle		0.77 (0.61, 1.00)		0.94 (0.72, 1.23)
Richer		0.54 (0.41, 0,72)		0.68 (0.50, 0.92)^**^
Richest		0.43 (0.31, 0,60)		0.53 (0.35, 0.80)^**^
**Number of gestations**				
Singletons		1		1
Multiple		1.99 (1.28, 3.08)		2.06 (1.33, 3.18)^**^
**Sex of child**				
Male		1		1
Female		0.80 (0.69, 0.92)		0.80 (0.69, 0.92)^**^
**Parity**				
Primiparous		1		1
Multiparous		0.87(0.69, 1.09)		0.86(0.68, 1.08)
Grand multiparous		0.96(0.73, 1.25)		0.98(0.75, 1.27)
**Toilet facility**				
Unimproved		1		1
Improved		1.06 (0.84,1.35)		1.07 (0.84, 1.36)
**Water source**				
Unimproved		1		1
Improved		1.05 (0.87, 1.26)		1.06 (0.88, 1.27)
**Place delivery**				
Home		1		1
Health facility		0.94 (0.79, 1.13)		0.94 (0.79, 1.13)
**Marital status**				
Never in union		1		1
Married		0.77 (0.28, 2.09)		0.72 (.027, 1.94)
Living with partner		0.87 (0.23, 3.23)		0.80 (0.23, 2.98)
Widowed		0.61 (0.18, 2,05)		0.62 (0.19, 2.08)
Divorced		0.73 (0.25, 2.13)		0.66 (0.22, 1.91)
No longer living together/separated		0.72 (0.22, 2.36)		0.70 (0.21, 2.29)
**Community level factors**				
**Residence**				
Urban			1	1
Rural			1.02 (0.75, 1.38)	0.72 (0.51, 1.00)
**Region**				
Tigray			1	1
Afar			0.78 (0.52, 1.19)	0.7 (0.46, 1.05)
Amhara			0.70 (0.47, 1.06)	0.66 (0.44, 0.98)^*^
Oromia			0.39 (0.26, 0.59)	0.35 (0.23, 0.51)^*^
Somali			0.65 (0.42, 1.01)	0.55 (0.36, 0.84)^**^
Benishangul			0.72 (0.48, 1.1)	0.64 (0.43, 0.97)^*^
SNNPR			0.54 (0.36, 0.8)	0.48 (0.33, 0.71)^**^
Gambella			0.38 (0.23, 0.60)	0.37 (0.24, 0.59)^**^
Harari			0.49 (0.31, 0.77)	0.52 (0.33, 0.81)^**^
Addis Abeba			0.18 (0.08, 0.34)	0.21 (0.11, 0.42)^**^
Dire Dawa			0.42 (0.26,0.7)	0.45 (0.28, 0.72)^**^
**Community maternal education**				
Low			1	1
High			0.86 (0.68, 1.09))	1.04 (0.82, 1.33)
**Community poverty status**				
Low			1	1
High			1.74 (1.37, 2.21)	1.44(1.11, 1.87)^*^
**Community level variance**				
Log likelihood	−2604.0147	−2532.0628	−2543.3866	−2504.4806
Deviance	5208.0294	5064.1256	5086.7732	5008.9612
MOR	1.7	1.673	1.58	1.51
PCV	Ref	0.41	0.52	0.61
ICC (%)	13	8	7	5

*Key: AOR: Adjusted odds ratio; CI: confidence interval; ICC: intra-cluster correlation; MOR: median odds ratio; p-value < 0.05* ; p-value < 0.01** *, PCV: Proportional Change in Variance.*

## Discussion

Undernutrition among under-five children in Ethiopia is a major public health problem. This study explored the spatial distribution and factors associated with coexisting forms of undernutrition among under-five children in Ethiopia. The spatial analysis result revealed that the spatial distribution of coexisting forms of undernutrition among under-five children was significantly varied across areas in Ethiopia. In the mixed effect binary logistic regression analysis, maternal education, household wealth status, sex of child and number of gestations, region, and community poverty status were found significantly associated with coexisting forms of undernutrition among under-five children.

The prevalence of a coexisting form of undernutrition among under-five children in Ethiopia was 19.61% (95% CI: 18.52, 20.74). It was lower than the prevalence reported in Cameroon [40] and Pakistan [41]. The difference could be attributed to variations in the study population and the operational definition of coexisting malnutrition. The study conducted in Cameroon focused exclusively on under-five children in rural areas, excluding urban children, which may have led to an overestimation of the prevalence of coexisting forms of undernutrition. In contrast, the study from Pakistan used a different operational definition that included overweight and obesity alongside stunting, wasting, and underweight, which may have contributed to the observed differences in prevalence.

The study revealed significant spatial variation in the coexisting forms of undernutrition among under-five children in Ethiopia. The higher risk observed in Tigray, Afar, Amhara, Benishangul-Gumuz, northern Oromia, and northeast Somali regions, as identified by the SaTScan analysis, could be attributed to multiple factors, including food insecurity, limited access to healthcare, poor maternal nutrition, and inadequate child feeding practices. These regions are often characterized by harsh climatic conditions, recurrent droughts, and socio-economic disparities [42,43], all of which contribute to a higher burden of undernutrition. Additionally, the identification of secondary clusters in the central and southern Somali regions suggests that malnutrition is also influenced by regional disparities in healthcare infrastructure, sanitation, and dietary diversity.

In the multilevel binary logistic regression analysis, children born to mothers who attained formal education had lower odds of coexisting forms of undernutrition compared to those born to mothers who had no formal education. It aligns with study findings reported in Bangladesh [44], Uganda [45], and Cameroon [40]. It could be due to educated mothers being more likely to adopt optimal breastfeeding and complementary feeding practices, ensure timely immunization, and seek medical care when needed, all of which contribute to better child nutrition and overall health [46,47]. In addition, education empowers mothers to make informed decisions regarding nutrition, hygiene, and healthcare access, ultimately improving children’s growth and development while mitigating the burden of malnutrition [48,49].

Household wealth status was significantly associated with coexisting forms of undernutrition among under-five children. Children who belonged to the richer and richest households had decreased odds of having coexisting form of undernutrition compared to the poorest households. Likewise, children belonging to a community with high poverty had higher odds of coexisting form of undernutrition. It was consistent with studies reported in sub-Saharan Africa [16], Cambodia [50] Bangladesh [51], and Gambia [52]. This association can be attributed to better access to nutritious food, improved healthcare services, higher maternal education, and overall better living conditions in wealthier households [53,54]. Adequate financial resources enable families to provide diverse and nutrient-rich diets, access clean water and sanitation, and seek timely medical care, all of which contribute to improved child nutrition [55,56]. The reduced risk in these populations highlights the critical role of socio-economic status in shaping child health outcomes and underscores the need for targeted interventions to support the most vulnerable households.

This study found that female children had significantly lower odds of experiencing coexisting forms of undernutrition compared to their male counterparts. This association is consistent with findings from previous studies conducted in similar settings [16,51]. Several biological and socio-cultural mechanisms may explain this sex-based disparity. From a biological perspective, female children are thought to possess greater physiological resilience to undernutrition, partly due to higher fat reserves and a lower basal metabolic rate, which may help buffer against energy and nutrient deficiencies during critical growth periods [57]. Additionally, females generally exhibit stronger immune responses in early childhood, potentially reducing susceptibility to infection-related malnutrition. Socio-culturally, while gender bias in health and nutrition often disadvantages girls, in certain Ethiopian communities, especially in rural areas, female children may paradoxically receive more consistent care, particularly in feeding and hygiene practices, as they are perceived to be more vulnerable or obedient, thereby eliciting greater maternal attention [49].

Another significant predictor of coexisting forms of undernutrition among under-five children was the number of gestations, with multiple births being at a higher risk compared to singletons. This increased risk may be due to several factors, including intrauterine growth restriction, lower birth weight, and higher nutritional demands among multiple births [58,59]. Additionally, mothers of multiples may face greater challenges in breastfeeding, resource allocation, and overall childcare, which can contribute to inadequate nutrition and higher susceptibility to stunting, wasting, and underweight in these children. Consistent with findings from previous studies, this analysis revealed that under-five children residing in communities with higher levels of poverty had significantly greater odds of experiencing coexisting forms of undernutrition compared to those in communities with lower poverty levels. This association may be explained by the fact that improved community-level socioeconomic conditions can enhance household access to adequate nutrition, promote optimal infant and young child feeding practices, and increase utilization of essential maternal and child health services, including facility-based deliveries. Furthermore, better socioeconomic environments may reduce exposure to infectious diseases, such as diarrheal illnesses, respiratory infections, and parasitic diseases, which are known to contribute to both acute and chronic forms of undernutrition. Therefore, addressing structural poverty hidden within the community level may benefit in reducing nutritional deficiency. Another important community-level factor identified in this study was the region of residence. Children from regions such as Oromia, SNNPR, Somali, Benishangul-Gumuz, Gambella, Harari, Addis Ababa, and Dire Dawa demonstrated significantly lower odds of experiencing coexisting forms of undernutrition compared to those from Tigray, with the most pronounced reduction observed in Addis Ababa.

The lower prevalence of undernutrition in Addis Ababa may reflect several factors. As the capital city, Addis Ababa benefits from better infrastructure, greater access to healthcare services, and improved urban planning, all of which contribute to favorable child health outcomes. Urban settings like Addis Ababa often offer greater food diversity, enhanced availability of fortified or nutrient-rich foods, and broader access to nutrition-specific programs and social protection schemes. Additionally, urban residents tend to have better access to information through mass media, health promotion campaigns, and educational opportunities, which may translate into improved caregiver knowledge and practices regarding child feeding, hygiene, and healthcare utilization.

These findings suggest that investments in infrastructure, health services, and social determinants of health in urban settings can contribute to reductions in undernutrition while highlighting the need to prioritize similar interventions in rural and underserved regions.

### Strengths and limitations of the study

This study has several methodological and analytical strengths. First, it utilizes a large, nationally representative, and weighted dataset, enhancing the generalizability of the findings to the broader population of Ethiopia. Second, the use of a mixed-effects logistic regression model appropriately accounts for clustering at different levels, improving the reliability of the estimates and standard errors. Third, advanced spatial analytical techniques including ArcGIS and SaTScan were applied to detect and visualize the spatial distribution and hotspots of coexisting forms of malnutrition. These spatial methods offer important insights into geographic disparities and spatial heterogeneity, thereby informing location-specific public health interventions and nutrition policies. Despite these strengths, the findings should be interpreted considering several limitations. First, the cross-sectional nature of the data precludes any inference of causality or temporal relationships between exposure and outcome. Second, the study relies on self-reported data, which may introduce reporting bias or social desirability bias, especially in culturally sensitive domains such as reproductive health, potentially affecting the accuracy of some variables. Third, although SaTScan is a powerful tool for detecting clusters, it uses a circular scanning window, which may not accurately capture the true shape and extent of irregularly shaped clusters, potentially limiting the precision of spatial boundaries.

## Conclusion

This study provides critical national-level evidence on the spatial distribution and determinants of coexisting forms of undernutrition among children under five in Ethiopia. The findings revealed marked spatial heterogeneity, with significant clustering in specific regions, underscoring the need for geographically targeted interventions. Key predictors including maternal education, household wealth status, child’s sex, region of residence, community-level wealth, and multiple gestations were significantly associated with the risk of coexisting undernutrition. These results emphasize the importance of integrated, multisectoral strategies that empower women through education, alleviate household poverty, and deliver tailored nutritional support to high-risk groups such as male children and those from multiple births. Public health practitioners and policymakers should leverage these insights to implement context-specific, equity-focused interventions aimed at reducing the burden of childhood undernutrition and breaking the cycle of intergenerational undernutrition.

### Implications of the findings

The findings of this study have important implications for public health policy and intervention strategies in Ethiopia. The high prevalence of coexisting forms of undernutrition among children under five particularly in regions such as Afar, Amhara, Tigray, Benishangul-Gumuz, and northern Somali underscores the urgent need for geographically targeted nutrition programs. The strong association between maternal education and household wealth with reduced odds of undernutrition highlights the importance of empowering women through education and improving socioeconomic conditions to combat child undernutrition. Moreover, the increased vulnerability observed among male children and those from multiple births indicates the need for tailored nutritional support for these high-risk groups. These insights can inform resource allocation and support the development of integrated, equity-focused interventions aimed at reducing undernutrition and promoting child health across Ethiopia’s diverse regions.
